# Surgical Outcome after Sleeve Pneumonectomy for Thoracic Malignancy: A Comparison Between Salvage and Non-salvage

**DOI:** 10.14789/jmj.JMJ23-0026-OA

**Published:** 2023-10-19

**Authors:** KOTA IMASHIMIZU, KENJI SUZUKI, SHINSUKE UCHIDA, MARIKO FUKUI, ARITOSHI HATTORI, TAKESHI MATSUNAGA, SHIAKI OH, KAZUYA TAKAMOCHI

**Affiliations:** 1 Department of General Thoracic Surgery, Juntendo University School of Medicine, Tokyo, Japan; 1 Department of General Thoracic Surgery, Juntendo University School of Medicine, Tokyo, Japan

**Keywords:** lung cancer, sleeve pneumonectomy, carinal resection, tracheo-bronchial anastomosis

## Abstract

**Objectives:**

Tumors invading the tracheobronchial angle or carina have long presented a challenge due to the complexity of airway reconstruction and management; thus, few medical centers have developed experience with this type of surgery. In this report, we review our experience with Sleeve Pneumonectomy (SP) and analyze both operative risks and outcomes.

**Materials and Methods:**

A retrospective review identified 34 patients who underwent SP: 19 underwent salvage SP and 15 underwent non-salvage SP. Salvage surgery was performed for recurrent lung cancer after chemoradiotherapy and could be considered if there were no other therapeutic options or in the presence of urgent symptoms, such as hemoptysis, obstructive pneumonia, superior vena cava syndrome, or tracheoesophageal fistula.

The perioperative morbidity and oncological outcomes of salvage and non-salvage SP were analyzed.

**Results:**

Most cases were of lung cancer, whereas salvage SP included one case of SVC syndrome due to metastasis of colon cancer and one case of hemoptysis due to metastasis of leiomyosarcoma. Complications occurred in 47% of the non-salvage SP cases and 53% of the salvage SP cases. The 30-day mortality rates were zero in the non-salvage cases and 11% in the salvage cases. The 90-day mortality rates were 20% and 16% in the non-salvage and salvage groups, respectively.

**Conclusions:**

The salvage of SP after chemoradiotherapy or in the presence of urgent symptoms is feasible. We believe that it can be an option that improves quality of life (QOL) through longer desease-free survival (DFS) and alleviation of symptoms, rather than waiting for tumor growth progression and exacerbation of symptoms.

## Introduction

Advancements in surgical and anesthetic techniques and postoperative management has enabled for the successful surgical resection of locally advanced lung cancers. In particular, tumors invading the tracheobronchial angle or carina have long presented challenges owing to the complexity of airway reconstruction and management. Moreover, sleeve pneumonectomy (SP) was performed in only 10 patients (9 with lung cancer and 1 with other tumors) according to the annual report of thoracic and cardiovascular surgeries in Japan in 2018^1)^; thus, few medical centers have developed experience with this type of surgery. In this report, we review our experiences with SP and analyze both the operative risks and outcomes.

## Material and methods

### Study population

Between May 2008 to March 2023, 34 SP were performed at our institution for centrally located lung cancer and metastatic malignant tumors. A retrospective data review was performed on the surgical outcomes and clinicopathological characteristics under a waiver of informed consent approved by the Juntendo University School of Medicine Institutional Review Board, Tokyo, Japan (IRB approval number: 23-0118). No data were missing for any of the variables used in this study.

### Indication, operative mode, and follow-up policy

When R0 was possible, PS was generally indicated for centrally located malignant tumors. Salvage surgery was performed for recurrent or persistent non-small cell lung cancer (NSCLC) after chemoradiotherapy (CRT) with curative intent in patients initially excluded from surgical resection. Surgery was considered if there were no other therapeutic options or in the presence of urgent symptoms, such as hemoptysis, obstructive pneumonia, superior vena cava (SVC) syndrome, or tracheoesophageal fistula. Patient selection is critical to obtain the positive benefits of a salvage surgery and careful re-staging should be performed before indicating a surgery as a salvage therapeutic option. Chest computed tomography (CT) and fluorodeoxyglucose (FDG)-positron emission tomography (PET) scans are essential for restaging when a relapse or recurrent disease is suspected. All clinical cases required discussions at multidisciplinary lung cancer meetings. In this series, all operative approaches were essentially performed via open thoracotomy because of the technicality of the procedures. All patients underwent double-lumen endotracheal intubation. The surgical approach used was a standard posterolateral thoracotomy. No irreversible procedures were performed before thoracic cavity exploration and confirmation of resectability by frozen-section analysis. If required, en bloc resection of the tumor and adjacent structures (SVC, brachiocephalic vein, azygos vein, esophagus, phrenic nerve, vagus nerve, and left atrium) was performed. The recurrent laryngeal and vagus nerves were routinely identified and preserved, if possible. All tracheobronchial anastomoses were end-to-end anastomoses. A continuous running suture (4-5 stitches) was applied to the deepest site of the bronchial stump using 3-0 nonabsorbable monofilament sutures (Prolene, Ethicon Inc.). The remainder of the anastomosis was performed using an interrupted suture with 4-0 nonabsorbable monofilament sutures, called a hybrid anastomosis^2, 3)^. Each suture was inserted through the full width of the bronchial wall, and all knots were tied outside of the lumen. The anastomosis was covered with a pericardial fat pad when needed, particularly in cases of severe diabetes mellitus, induction chemotherapy, or chemoradiotherapy. A chin stitch was frequently used to guide the trachea toward the periphery. When resection and reconstruction of the SVC were necessary, concomitant vascular reconstruction was performed with an expanded polytetrafluoroethylene (ePTFE) graft and 5-0 nonabsorbable monofilament sutures (Prolene, Ethicon Inc.). After pulmonary resection, a bronchoscopic examination was conducted on postoperative day 7 or before discharge. Routine bronchoscopic examinations were planned on postoperative days 14 and 28 and a grading sequence was used to describe bronchial healing^4)^.

## Results

**Figure 1:**
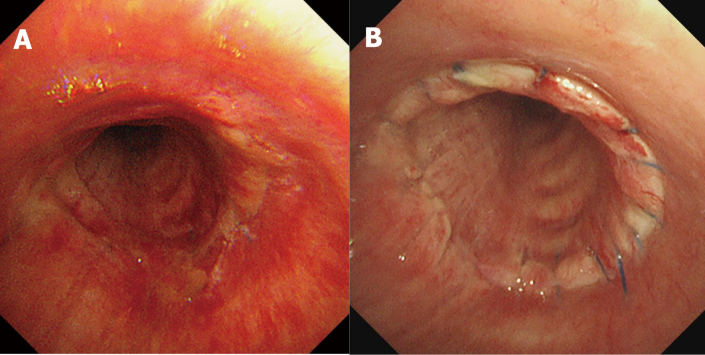
Case 31 underwent salvage sleeve pneumonectomy after chemoradiotherapy. Bronchoscopy showing healing of the tracheobronchial anastomosis on postoperative day 6 (A) and 55 (B).

Table 1 summarizes the surgery types, clinicopathological features, and outcomes of the 34 patients who underwent SP. Nineteen patients underwent salvage surgery: no intraoperative mortalities were observed. Of the 34 cases, 32 were on the right side and approached by standard posterolateral thoracotomy. The majority of cases were lung cancer, whereas salvage SP included one case of SVC syndrome due to metastasis of colon cancer and one case of hemoptysis due to metastasis of leiomyosarcoma. In case 2, a left pneumonectomy was performed using standard posterolateral thoracotomy. Because the bronchial stump was diagnosed with a positive resection margin by intraoperative rapid pathological diagnosis, the patient was repositioned, the carina was resected with a median sternotomy, and the trachea and right main bronchus were anastomosed. In contrast, in case 12, left sleeve pneumonectomy was performed using standard posterolateral thoracotomy without median sternotomy. Case 1 was a 75 mm large cell neuroendocrine carcinoma to the right of segment 6. Pneumonectomy was performed because the tumor had spread to the right main bronchus; however, a sleeve operation was required because of air leakage from the bronchial stump. Lymph node #7 was swollen to 72 mm and macroscopically N2, but pathologically negative. In case 19, a lesion was present in the lumen of the right main bronchus from B6. It was 5 mm away from the carina. Although there was no invasion at the tracheal bifurcation, SP was required because there was insufficient distance to close the bronchial stump. We identified the tumor as T1 as described in the TNM classification of lung carcinomas, “The uncommon superficial spreading tumor of any size with its invasive component limited to the bronchial wall is classified as T1.” Case 21 was judged inoperable by another hospital and was scheduled for chemotherapy after radiation therapy. However, before chemotherapy, the patient visited our hospital for a second opinion and underwent salvage surgery. Hilar lung cancer in case 24 was discovered due to dysphagia and coughing. The patient was diagnosed with Stage IIIB disease at the hospital of origin and received chemoradiotherapy followed by durvalumab. The patient was referred to our hospital with an esophageal-tracheal fistula. Right sleeve pneumonectomy with esophagectomy, cervical esophagostomy, and enterostomy was performed. Four days after the operation, a tracheobronchial anastomotic fistula developed, and reoperation was performed. We excised the old anastomotic site, anastomosed the trachea and bronchus at the new site, and covered it with the omentum. Although there was no problem with the anastomotic site, respiratory failure subsequently occurred, and the patient was lost on postoperative day (POD) 18 after the first operation. Postoperative pathology revealed no tumor cells after chemoradiotherapy. Patient 31 underwent sleeve right upper lobectomy by a previous doctor and was administered cisplatin and vinorelbine as adjuvant chemotherapy. Subsequently, local recurrence was diagnosed and chemoradiotherapy and then durvalumab were administered. Pembrolizumab was administered because it was diagnosed as recurrence by PET/CT. However, the tumor grew and the patient was referred to our hospital. Salvage SP was performed, and the patient was discharged without complications. Bronchoscopy revealed healing of the tracheobronchial anastomosis on POD 6 and 55 (Figure 1) without any problems, even after chemoradiotherapy.

**Table 1:** Summary of sleeve pneumonectomy cases

Patient	Indications	Side for surgery	Age/sex	Histopathology	Stage	Preoperative therapy	Reason for salvage surgery	Complications	Outcomes (months)
1	non-salvage	Right	60/M	LCNEC	IIB	-	-	-	Cancer death (3)
2	non-salvage	Left	69/M	Adenocarcinoma	IIIA	-	-	-	Cancer death (21)
3	salvage	Right	73/M	Squamous cell carcinoma	IIB	CRT	Hemoptysis	Af	Cancer death (4)
4	non-salvage	Right	66/M	Squamous cell carcinoma	IIIB	-	-	-	ADf (159)
5	non-salvage	Right	44/M	Adenocarcinoma	IIIB	-	-	Sinus tachycardia	Cancer death (3)
6	non-salvage	Right	43/F	Adenoid cystic carcinoma	IIIB	-	-	b.p fistula	Cancer death (11)
7	salvage	Right	58/M	Small cell carcinoma	IIB	CRT	Obstructive pneumonia	-	Cancer death (2)
8	salvage	Right	48/M	Metastatic colon carcinoma	-	chemotherapy	SVC syndrome	-	Cancer death (12)
9	non-salvage	Right	48/M	Squamous cell carcinoma	IIIB	-	-	-	ADf (136)
10	non-salvage	Right	67/M	Adenoid cystic carcinoma	IIIB	-	-	Af	Cancer death (7)
11	non-salvage	Right	66/M	Squamous cell carcinoma	IIIA	-	-	b.p fistula Empyema Pneumonia	exitus due to respiratory failure (2)
12	non-salvage	Left	78/F	Adenoid cystic carcinoma	IIIA	-	-	-	Cancer death (54)
13	non-salvage	Right	73/M	Pleomorphic carcinoma	IIIA	-	-	-	ADf (104)
14	salvage	Right	56/F	Metastatic leiomyosarcoma	-	-	Hemoptysis Obstructive pneumonia	Af	Cancer death (4)
15	salvage	Right	70/M	Adenocarcinoma	IIIB	-	SVC syndrome	Af	Cancer death (9)
16	non-salvage	Right	58/M	Squamous cell carcinoma	IIIA	-	-	b.p fistula Af	exitus due to pneumonia (42)
17	salvage	Right	66/M	Squamous cell carcinoma	IIB	CRT	Progressive disease	Af	Cancer death (12)
18	salvage	Right	47/M	Pleomorphic carcinoma	IIIB	-	t.e fistula	b.p fistula Empyema	Cancer death (6)
19	non-salvage	Right	57/M	Squamous cell carcinoma	IA	-	-	-	ADf (73)
20	non-salvage	Right	65/M	Squamous cell carcinoma	IIIA	-	-	Pneumonia	Cancer death (7)
21*	salvage	Right	65/M	Squamous cell carcinoma	IIIA	RT	stable disease	-	ADf (58)
22	salvage	Right	48/M	Squamous cell carcinoma	IIIA	CRT	t.e fistula post CRT	-	ADf (56)
23	salvage	Right	70/M	Squamous cell carcinoma	Rec.	CRT	Obstructive pneumonia	b.p fistula Af Empyema Anastomotic stenosis	exitus due to respiratory failure (43)
24	salvage	Right	45/M	P/D Carcinoma	-	CRT	t.e fistula post CRT	b.p fistula Empyema	exitus due to respiratory failure (1)
25	non-salvage	Right	77/M	Combined small cell carcinoma and squamous cell carcinoma	IIIB	-	-	b.p fistula	ADf (14)
26	salvage	Right	78/M	Squamous cell carcinoma	IIB	-	Obstructive pneumonia	-	Cancer death (6)
27	non-salvage	Right	56/M	Squamous cell carcinoma	IIIB	-	-	-	ADf (12)
28	salvage	Right	64/M	Pleomorphic carcinoma	IIIA	-	SVC syndrome	AE-IP	Cancer death (6)
29	salvage	Right	70/F	Adenocarcinoma	IVA	CRT	Radiation pneumonitis	b.p fistula AE-IP	exitus due to respiratory failure (1)
30	salvage	Right	72/M	Squamous cell carcinoma	IIA	CRT+ICI	ILD related ICI	AE-IP Anastomotic stenosis	exitus due to Anastomotic stenosis (6)
31	salvage	Right	67/M	Squamous cell carcinoma	Rec.	CRT+ICI	Local recurrence	-	ADf (8)
32	salvage	Right	62/F	Squamous cell carcinoma	IIIB	CRT+ICI	Progressive disease	-	ADf (5)
33	salvage	Right	67/M	Squamous cell carcinoma	Rec.	CRT+ICI	Local recurrence	-	ADf (1)
34	salvage	Right	43/M	Squamous cell carcinoma	Rec.	Lobectomy	Local recurrence	-	ADf (1)

*This patient underwent SP as salvage therapy. LCNEC, large cell neuroendocrine carcinoma; Rec, recurrence; CRT, chemoradiotherapy; RT, radiotherapy; ICI, immune checkpoint inhibitor; SVC syndrome, Superior vena cava syndrome; t.e fistula, tracheoesophageal fistula; ILD, Interstitial lung disease; b.p fistula, bronchopleural fistula; AE-IP, acute exacerbation of interstitial pneumonia; Af, Atrial fibrillation; ADf, Alive and disease-free; P/D, poorly differentiated.

Table 2 summarizes the comparison of the characteristics between salvage and non-salvage SP. In the non-salvage SP group, 13 (87%) of patients were men with an average age of 63.1 years and 13 (87%) underwent surgery on the right side. In the salvage surgery group, 16 (84%) patients were men with an average age of 61.5 years; all patients underwent surgery on the right side. Distant metastases were not observed in either group. The T-factors of salvage surgery were unreported in certain pathological reports because the salvage group included metastatic leiomyosarcoma, metastatic colon carcinoma, post-CRT cases, and recurrence cases. In cases of non-salvage SP, only the SVC and left atrium were resected. However, in the salvage surgery cases, there were additional cases of combined resection of the esophagus, left brachiocephalic vein, and azygos vein. No intraoperative mortality occurred. In the salvage group the 30-day mortality was 11% (2 of 19): respiratory failure after bronchopleural fistula, 1; respiratory failure after bronchopleural fistula and acute exacerbation of interstitial pneumonia, 1.

**Table 2:** Characteristics of the patients

Characteristics	Non-salvage (N=15)	Salvage (N=19)
Age	43-78 (63.1)	43-78 (61.5)
Sex		
Female	2	3
Male	13	16
Side		
Left	2	-
Right	13	19
Comorbidities		
Diabetes	2	1
Hypertension	1	2
Cerebral infarction	2	-
Arrhythmia	1	-
Chronic kidney disease		1
Dyslipidemia	1	1
Post PCI		1
T status		
1	-	- *
2	1	1*
3	4	3*
4	10	6*
N status		
0	4	7*
1	2	5*
2	9	5*
M status		
0	15	19
1	-	-
Pathology		
Squamous cell carcinoma	7	11
Adenocarcinoma	2	2
Pleomorphic carcinoma	1	2
Adenoid cystic carcinoma	2	-
Other	3	4
Combined resection		
Superior vena cava	1	7
Left atrium	2	1
Esophagus	-	3
Left brachiocephalic vein	-	2
Azygos vein	-	1
Preoperative treatment		
Chemoradiotherapy	-	11
Radiotherapy	-	1
Chemotherapy	-	1
Morbidity	7 (47%)	10 (53%)
Mortality 30 day	-	2 (11%)
Mortality 90 day	3 (20%)	3 (16%)

PCI: percutaneous coronary intervention * The T-factors and the N-factors of salvage surgery were not reported in some pathological reports because the salvage group included metastatic leiomyosarcoma, metastatic colon carcinoma, post-CRT cases, and recurrence cases.

Table 3 summarizes the postoperative morbidity between the salvage and non-salvage SP groups. Complications occurred in 47% of the non-salvage SP and 53% of the salvage SP cases. Bronchopleural fistula occurred in 27% of the non-salvage SP and 21% of salvage SP cases. In contrast, anastomotic stenosis was only observed in the salvage group (11%; 2 of 19).

**Table 3:** Postoperative morbidity

Complications	Non-salvage (N=15)	Salvage (N=19)
Total	7 (47%)	10 (53%)
Arrhythmia	3 (20%)	5 (26%)
Bronchopleural fistula:	4 (27%)	4 (21%)
Empyema	1 (7%)	2 (11%)
Pneumonia	2 (13%)	-
Anastomotic stenosis	-	2 (11%)
Acute exacerbation of interstitial lung disease	-	2 (11%)
(Broncho-pleural fistula related)	-	1 (5%)

## Discussion

Indications for sleeve SP are relatively uncommon and have been largely applied solely at specialized thoracic surgery centers. This lack of experience has led to unfamiliarity with the indications and results of tracheobronchial resection, frequently preventing it from consideration as a viable treatment option. Indeed, these tumors are often considered “unresectable” and treated with chemotherapy or chemoradiotherapy. Despite the complex technical challenges and risks associated with carinal resection, it offers patients a chance for long-term survival when the alternatives are simply palliative^5)^. Furthermore, salvage surgery—that is, surgery for recurrence or tracheoesophageal fistula after definitive CRT—is technically demanding and can lead to serious anastomotic complications. However, preoperative thoracic radiation is no longer considered a contraindication to resection^6)^. Our institute previously reported salvage surgery after definitive CRT in 27 patients with NSCLC. The median administered radiation dose was 60 Gy. Pneumonectomy was performed in nine patients, including two carinal resections, and lobectomy was performed in 18 patients, including five bronchoplasties. Although arrhythmia was observed more frequently in patients who underwent bronchoplasty, bronchopleural fistulas were found in only two patients who underwent pneumonectomy. Salvage surgery after definitive CRT is acceptable for NSCLC. Bronchoplasty or pneumonectomy should be considered as options, even after the administration of high-dose CRT^7)^.

Case 22 was previously reported in detail as a case report. Tracheoesophageal fistulae are among the most serious complications that occur during curative chemoradiotherapy for advanced lung cancer. Generally, tracheoesophageal fistulas have extremely poor prognosis, and most treatments are palliative; however, the patient recovered without major postoperative complications^8)^.

The overall morbidity after carinal resection or sleeve pneumonectomy ranges from 11% to 50%^9)^. Atrial fibrillation (AF) was seen in 17 cases and is a common complication after pneumonectomy that is relatively benign, easy to treat, and typically reversible. Mansour et al. reported no adverse effects from AF in patients with this complication. AF has not been found to result in prolonged postoperative hospitalization^10)^. One of the most challenging complications is acute respiratory distress syndrome (ARDS), which occurs in up to 20% of patients and has an associated mortality of 50-100%. The incidence of bronchopleural fistula (BPF) in carinal sleeve resections is generally within the range of 3.8-21.6% in the literature. BPF is a serious complication and is among the most common causes of morbidity and mortality^11)^. Post-pneumonectomy BPF is seen more often after right pneumonectomy and is clinically more severe than that observed after a lobectomy, with a mortality rate ranging from 25 to 71%^12, 13)^. Typically used treatment methods include tube thoracostomy, open window thoracostomy, thoracomyoplasty, closure of the fistula with rethoracotomy and reinforcing the stump with live autologous flaps, and transpericardial closure of the fistula with sternotomy^11-14)^. We encountered an anastomotic fistula in eight cases. One patient underwent chest drainage, but was subsequently lost due to acute exacerbation of interstitial pneumonia. Seven cases recovered with surgery: fenestration, or re-suturing and covering with an intercostal muscle flap or anterior mediastinal fat tissue. Some salvage cases had a poor prognosis; however, most of the deaths were due to cancer, and there were no problems with the indications for surgery. A notable feature of our study was the high frequency of salvage SP after recurrence, definitive CRT, and symptoms such as hemoptysis that must be treated. It has been reported that the mortality rate of pneumonectomy after CRT is 24% in North America and is considered almost contraindicated^15)^. Case 31 was a salvage case for recurrence after postoperative CRT. As mentioned previously, no complications occurred at the anastomotic site. Therefore, post- CRT did not result in surgery not occurring. Non-salvage cases are treated to achieve a complete cure; however, salvage cases include progressive diseases after definitive CRT, hemoptysis, obstructive pneumonia, SVC syndrome, and tracheoesophageal fistulas post-CRT. As such, no other treatment options are currently available. Morbidity was relatively high (47%, non-salvage; 53%, salvage), but in our study the 30-day mortality was zero for non-salvage and 11% for salvage. We believe that it is an option for improving quality of life (QOL) through longer desease-free survival (DFS) and alleviation of symptoms rather than waiting for tumor growth progression and exacerbation of symptoms.

This study was limited by its single-center retrospective design. Additionally, only patients who underwent surgical resection were enrolled. Almost all patients in our study were initially treated at different institutions and were selected for the evaluation of salvage surgery for different reasons, reflecting the heterogeneity of the population and treatment approaches. Nevertheless, this study provides new information regarding the surgical options for salvage sleeve pneumonectomy. Further studies with larger sample sizes are required.

## Conclusions

Further research is necessary to determine appropriate indications for this challenging procedure; however, salvage sleeve pneumonectomy after chemoradiotherapy or in the presence of urgent symptoms is feasible with acceptable mortality and morbidity and promising long-term results.

## Funding

This work was supported in part by a Grant-in-Aid for Cancer Research from the Ministry of Health, Labor and Welfare of Japan; the Smoking Research Foundation; and the National Cancer Center Research and Development Fund (26-A-4).

## Author contributions

All authors read and approved the final manuscript.

## Conflicts of interest statement

The authors declares that there are no conflicts of interest.
